# A novel design of bioartificial kidneys with improved cell performance and haemocompatibility

**DOI:** 10.1111/jcmm.12029

**Published:** 2013-03-11

**Authors:** Zay Yar Oo, Karthikeyan Kandasamy, Farah Tasnim, Daniele Zink

**Affiliations:** Institute of Bioengineering and NanotechnologyThe Nanos, Singapore

**Keywords:** Bioreactor, hollow fibre membrane, haemocompatibility, human primary renal proximal tubular cells, interleukin, immunomodulation

## Abstract

Treatment with bioartificial kidneys had beneficial effects in animal experiments and improved survival of critically ill patients with acute kidney injury in a Phase II clinical trial. However, a Phase II b clinical trial failed. This and other results suggested various problems with the current design of bioartificial kidneys. We propose a novel design to improve various properties of device, including haemocompatibility and cell performance. An important feature of the novel design is confinement of the blood to the lumina of the hollow fibre membranes. This avoids exposure of the blood to the non-haemocompatible outer surfaces of hollow fibre membranes, which usually occurs in bioartificial kidneys. We use these outer surfaces as substrate for cell growth. Our results show that commercial hollow fibre membranes can be directly applied in the bioreactor when human primary renal proximal tubular cells are grown in this configuration, and no coatings are required for the formation of robust and functional renal epithelia. Furthermore, we demonstrate that the bioreactor unit produces significant amounts of interleukins. This result helps to understand the immunomodulatory effects of bioartificial kidneys, which have been observed previously. The novel bioartificial kidney design outlined here and the results obtained would be expected to improve the safety and performance of bioartificial kidneys and to contribute to a better understanding of their effects.

## Introduction

Bioartificial kidneys (BAK) combine a haemofilter in series with a bioreactor. Animal studies 1–7 and clinical trials 8, 9 were performed with BAK where both units consisted of commercial haemodialysis/haemofiltration cartridges. The cartridges contained hollow fibre membranes (HFM) and renal proximal tubular cells resided on the luminal surfaces in the bioreactor unit. Both, the blood and the haemofiltrate were directed from the haemofilter into the bioreactor. The haemofiltrate flew in the lumina of the HFM in the bioreactor, where it was directly exposed to the cells. The blood was directed to the extra-HFM space, where it was separated from the renal cells by the semi-permeable membrane. This architecture mimicked the architecture of the renal proximal tubule. It was expected that the bioreactor unit would replace functions of the renal proximal tubule.

In a Phase I/II clinical trial no clear indication could be found that proximal tubular-specific functions were contributed by the renal cells 8, 10. Improved long-term survival in the group of patients that received BAK treatment was observed in a Phase II clinical trial, which enrolled critically ill patients with acute kidney injury (AKI) 9. The reasons for this remained unclear 10, 11. Cytokine levels in serum and plasma were measured in various animal studies and the Phase I/II clinical trial 1, 2, 5–8. Some changes in cytokine levels suggested that beneficial effects of BAK treatment might be a result of immunomodulatory functions 1, 2, 5–8. However, the release of cytokines or other immunomodulators from the bioreactor unit has not been investigated, so far. A Phase II b clinical trial failed in 2006 (discussed in 9), and since then no further clinical trials with BAK have been performed.

There are various problems with the BAK design outlined above, which has been applied now for more than a decade. Major problems are related to the fact that the blood normally flows in the lumen of the HFM in haemodialysis/haemofiltration cartridges during renal replacement therapy. Thus, the inner HFM surfaces are usually smooth and optimized for haemocompatibility, in contrast to the outer surfaces, which are often rough with large pores. Haemocompatibility can be compromised when the blood is exposed to these rough outer surfaces in the bioreactor unit. Additional problems are related to irreversible clogging of the pores on the outer surface 12.

Furthermore, so far only human primary renal proximal tubular cells (HPTC) have been approved for clinical trials of BAK 4, and this cell type does not perform well on smooth and haemocompatible surfaces of synthetic membranes 10, 12–15. To address this problem, protein coatings have been applied to the luminal surfaces of the HFM in BAK bioreactor units 1–9. More recent data suggested that single-layered protein coatings might not be sufficient and that double coatings consisting of 3,4-dihydroxy-L- phenylalanine (DOPA) and collagen IV might be preferable 13. However, a better solution would be the use of HPTC-compatible membrane surfaces that do not require any coatings.

Other problems with the BAK design outlined above are related to the fact that all or a substantial fraction of the blood flows through both units 1–9, and would be exposed to the membrane surfaces in both cartridges. This further compromises haemocompatibility. As outlined, the blood and the haemofiltrate flow in the bioreactor unit on opposite sides of the HFM. The flow rate of the haemofiltrate is much lower than the flow rate of the blood (for instance, 10 ml/min. *vs* 150 ml/min. in 8), when all 3, 6 or a substantial fraction 1, 2, 4, 7–9 of the blood exiting the haemofilter flows into the bioreactor. This leads to generation of transmembrane pressure. In one study, the pressure in the haemofiltrate line was 5–10 mm Hg, whereas it was 10–25 mm Hg in the blood line 3 (no values provided in other studies). Renal epithelial cells are highly sensitive to transmembrane pressure, and pressure differences destroy the epithelia in bioreactors 16, 17. Therefore, differences in the flow rates of the haemofiltrate and the blood must be avoided in the bioreactor unit.

Here, we developed an alternative BAK design to eliminate the problems outlined above. One major feature of this design is that HPTC grow on the outsides of HFM in the bioreactor unit. HPTC performance on the outer surfaces of unmodified and uncoated commercial HFM was investigated under bioreactor conditions. Furthermore, the release of interleukins from the bioreactor was studied to address potential immunomodulatory functions.

## Materials and methods

### Cell culture

Human primary renal proximal tubular cells, the porcine proximal tubular-derived cell line LLC-PK1 and the murine fibroblast cell line NIH 3T3 were obtained from the American Type Culture Collection (ATCC, Rockville, MD, USA) and cultivated as described 12, 18. HPTC were used up to passage 5.

### Static culture and double coating of HFM

Highflux polyarylethersulfone (PAES) HFM were obtained from the haemofilter of the PrismafleX HF20 set (Gambro Singapore, Singapore) and highflux polysulfone (PSF) HFM were derived from the HF80S haemofilter (Fresenius Medical Care, Bad Homburg, Germany). HFM consisting of polyethersulfone/polyvinylpyrrolidone (PES/PVP) were produced as described 12. HFM and glass capillaries (Sutter Instrument, Novato, CA, USA) were sterilized with 70% ethanol and UV irradiation in 24-well tissue culture plates (Nunc, Naperville, IL, USA) and were subsequently washed with phosphate-buffered saline (PBS). After cell seeding the samples were gently agitated for 4 hrs on a shaker that was placed in an incubator. Cell culture medium was changed on the following day and the cells were cultivated for 3 days. In some experiments the outer surfaces of the HFM were double coated with DOPA and collagen IV as described 12, 13.

### Scanning electron microscopy (SEM), immunostaining and determination of γ-glutamyl transferase (GGT) activity

These methods were performed as described 12.

### Quantitative real-time polymerase chain reaction (qPCR)

Quantitative real-time polymerase chain reaction (qPCR) was performed as described 12, 19.

### HFM bioreactors

Highflux PAES HFM with an inner diameter of 215 μm and a wall thickness of 50 μm (Gambro Singapore) were applied in all bioreactors. Small bioreactors containing 1 single HFM and medium-sized bioreactors with 25 HFM were constructed with polypropylene housings made from 1 ml syringes (B. Braun Melsungen AG, Melsungen, Germany). Larger bioreactors containing 250 HFM were constructed with a polypropylene housing made from 10 ml syringes. HFM were glued to gas-permeable tubings (PharMed BPT tubing, Cole-Parmer, Vernon Hills, Illinois, USA) in the small and medium-sized bioreactors for luminal perfusion. For construction of the larger bioreactors luer-lock tips of 5 ml syringes were glued to the HFM for luminal perfusion. HFM connected to tubings or luer-lock tips were glued together with gas-permeable tubings for extra-HFM perfusion into the polypropylene housings. Three-way male lock stopcocks were inserted into the inlet and outlet tubings of intra- and extra-HFM circuits for sample collection and cell seeding. Perfusion was driven by a multi-channel peristaltic pump (Ismatec, Glattbrugg, Switzerland).

### HFM bioreactor handling and cell seeding

Before cell seeding HFM bioreactors were perfused (1 ml/min.) with 70% ethanol for 8 hrs and subsequently with sterile PBS overnight. The cell suspension with 3 × 10^6^–5 × 10^6^ cells/ml was injected into the extra-HFM space. The following volumes of cell suspension were used for cell seeding: 600 μl (25-HFM bioreactor), 4.0–4.5 ml (250-HFM bioreactor) and 30–35 ml (commercial haemofilter). After cell seeding the bioreactor was rotated by 90 degrees every 2 hrs and rotation was performed three times. Perfusion started 2 hrs after the last rotation and was performed with cell culture medium at a flow rate of 80 μl/min. unless indicated otherwise. Perfusion was continued for 7 days before the assays were performed.

### Lucifer yellow uptake and transport

The extra-HFM space of HPTC-containing 25-HFM bioreactors was perfused with normal cell culture medium, whereas HFM lumina were perfused with cell culture medium containing 80 μM of lucifer yellow. Perfusion was performed for 24 hrs at flow rates of 80 μl/min. The same conditions were applied to 25-HFM cartridges without cells that were used as controls. After 24 hrs of perfusion samples from the extra-HFM space were collected. The fluorescence intensity was measured by using a microplate reader (Tecan Safire2™, Mannedorf, Switzerland) with excitation and emission wavelengths of 428 and 540 nm respectively. To assess cellular uptake of lucifer yellow HFM derived from the bioreactors were fixed with formaldehyde (3.7% in PBS) and stained with 4′,6-diamidino-2-phenylindole (DAPI) for epifluorescence microscopy. From some of the HFM, the cells were detached with trypsin-versene, fixed with formaldehyde and stained with DAPI. Epifluorescence imaging was performed by using an Olympus BX-DSU microscope (Olympus, Tokyo, Japan).

### Determination of the back-leak of urea and creatinine

Extra-HFM spaces of 25-HFM bioreactors were perfused with cell culture medium containing 2 mg/ml of urea (Invitrogen, Singapore) and 0.1 mg/ml of creatinine (Sigma-Aldrich, Singapore). The luminal spaces were perfused with normal cell culture medium without urea and creatinine. Perfusion was performed for 4 hrs at flow rates of 80 μl/min. Samples from both compartments were collected and the concentrations of urea and creatinine were measured with an i-STAT analyser (Abbott Point of Care Inc., Princeton, NJ, USA).

### Cytokine release

The extra-HFM space of 25-HFM bioreactors was perfused with cell culture medium containing 10 μg/ml of lipopolysaccharides (LPS) from *Escherichia coli* (Sigma-Aldrich). Control bioreactors were perfused with normal cell culture medium. The bioreactors were perfused for 24 hrs at flow rates of 80 μl/min. Interleukin-6 (IL-6), IL-8 and IL-10 levels in the cell culture medium were determined after 24 hrs by using human IL-6, IL-8 and IL-10 ELISA kits (Life Technologies, Singapore).

### Exposure to ultrafiltrate with a relevant perfusion rate

Ultrafiltrate was prepared from inactivated human serum (Invitrogen) by using Vivaspin 20 ultrafiltration devices (Sartorius AG, Goettingen, Germany) with a molecular weight cut-off of 30 kD. The extra-HFM space of 25-HFM bioreactors was perfused with the ultrafiltrate at a flow rate of 0.5 ml/min. for 16 hrs. At this flow rate, velocity and shear stress on the cells in a 25-HFM bioreactor are the same as in a PrismafleX HF20 haemofilter when a flow rate of 10 ml/min. is applied. After 16 hrs cell performance was assessed by epifluorescence microscopy, immunostaining and determination γ-glutamyl transferase (GGT) activity.

### Statistics

All statistical analyses were performed using the SigmaStat (3.5) software (Systat Software Inc., Chicago, IL, USA). All the data were tested for normal distribution and the test was passed in all cases. The student's two-tailed *t*-test was used for determining significance levels and at least three replicates were included in each experiment. Significant differences (*P* < 0.05) are marked with an asterisk in the graphs.

## Results

### Novel BAK design

**Fig. 1:**
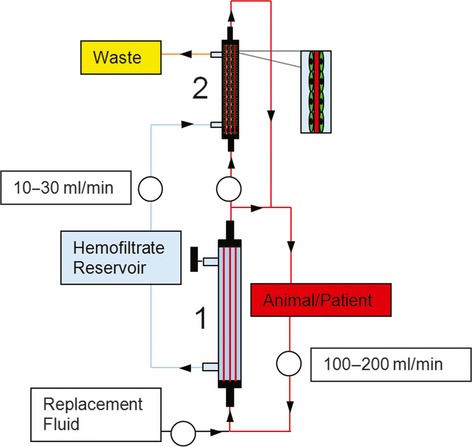
BAK design. The blood flows from the animal or patient (red, bloodline: red) at a flow rate of 100–200 ml/min. into the haemofilter (1), which consists of a normal haemodialysis/haemofiltration cartridge. The drawing illustrates the pre-dilution mode and pumps are symbolized by circles. The extra-HFM space containing the haemofiltrate and the haemofiltrate reservoir are depicted in blue. The blood outlet port of the haemofilter is linked to a 3-way connector and most of the blood flows back to the animal/patient. The remaining blood and the haemofiltrate are pumped at similar flow rates of ∼10–30 ml/min. into the bioreactor (2). HPTC (green) grow in the bioreactor on the outer surfaces of the HFM, where they are exposed to the haemofiltrate. The blood flows in the lumina of the HFM. This configuration is shown enlarged in the upper right. The processed haemofiltrate exiting the bioreactor is depicted in yellow (waste).

We developed a novel BAK design for large animal and clinical studies, which is illustrated in Figure 1. As usual, the first unit is a normal commercial haemofilter, and the blood flow rate in this unit would be in the normal range of 100–200 ml/min. Under these conditions ∼10–30 ml haemofiltrate/min. are obtained. The haemofiltrate, as well as blood exiting the haemofilter, would flow into the bioreactor. In contrast to previous approaches, the flow rates of the blood and of the haemofiltrate entering the bioreactor would be the same and in the range of 10–30 ml/min. This would eliminate the problem of transmembrane pressure in the bioreactor.

As the flow rate of the blood in the bioreactor would be relatively low in comparison to the haemofilter, most of the blood exiting the haemofilter would be directed back to the animal/patient and would not flow into the bioreactor. Thus, in contrast to previous approaches, most of the blood would not be exposed to both cartridges, which would improve haemocompatibility. Furthermore, this kind of set-up allows the bloodline to discontinue to the bioreactor rapidly in case of problems. This would improve the safety of the device.

In contrast to previous approaches, the blood would flow in the bioreactor in the HFM lumen. This would ensure that the blood is always exposed to the haemocompatible inner surfaces of the HFM. The renal cells would grow on the outsides of the HFM and would be exposed to the haemofiltrate flowing in the extra-HFM space. As HPTC performance appears to be compromised on haemocompatible membrane surfaces it would be expected that cell performance would be improved on the outer non-haemocompatible HFM surfaces. Therefore, we investigated HPTC performance on the outer surfaces of commercial HFM and assessed whether under these conditions membrane coatings were still required.

### HPTC performance on the outer surfaces of unmodified commercial HFM

**Fig. 2:**
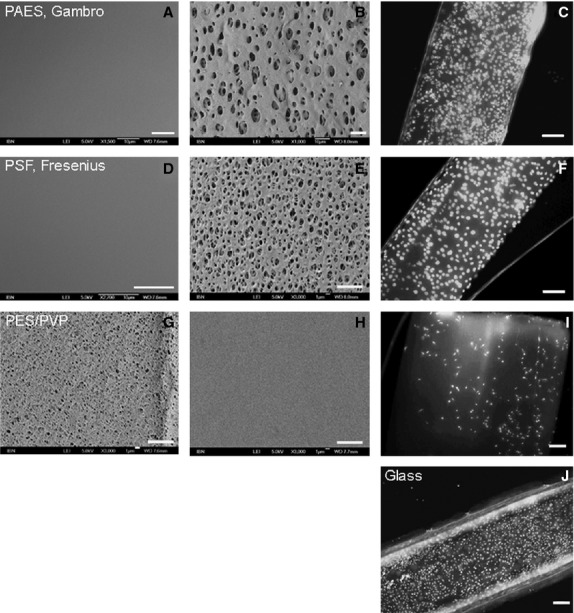
Cell growth on HFM. Each row shows SEM images of the inner (left-hand panels; **A**, **D** and **G**) and the outer (middle; **B**, **E** and **H**) surface of HFM and an epifluorescence image of the DAPI-stained nuclei (white spots) of HPTC grown on the outer surface of the respective HFM (**C**, **F** and **I**). A glass capillary (positive control) with HPTC grown on the outer surface (DAPI-stained nuclei: white spots) is shown in (**J**). The different rows show HFM consisting of PAES (Gambro; A, B and C), PSF (Fresenius; D, E and F) and PES/PVP (non-commercial, G, H and I). HFM consisting of PES/PVP (outer diameter: 780 μm) were used as negative control and only few cells survived on this material (I), whereas confluent monolayers were formed on the outer surfaces of all other materials (C, F and J). The inner surfaces of the commercial HFM consist of the smooth skin layer (A and D), whereas their outer surfaces are rough with relatively large pores (B and E). The PES/PVP HFM have a rough and microporous inner surface (G) and the smooth skin layer is on the outer surface (H) 12. Scale bars: A, B and D: 10 μm; E, G and H: 5 μm; C and F: 100 μm; I and J: 200 μm (HFM were squeezed and flattened for epifluorescence microscopy).

For addressing HPTC performance on the outer surfaces of HFM we used HFM consisting of PSF or PAES. These HFM, which were derived from commercial haemofilters, showed the typical architecture and the inner surface consisted of the smooth skin layer, whereas the outer surface was rough with relatively large pores (Fig. 2A, B, D and E). Unmodified commercial HFM that were not coated before cell seeding were used in the following experiments.

In a first set of experiments, HPTC were grown on the outer surfaces of the HFM in static culture. After 3 days, a confluent epithelium was formed (Fig. 2C and F). A similar epithelium was obtained with glass capillaries, which served as positive control (Fig. 2J). We showed before that unmodified glass is an excellent substrate for HPTC 14, 20. In contrast, only few cells were observed on HFM consisting of PES/PVP (Fig. 2I), which were used as negative control. The PES/PVP HFM used here have a reversed architecture and the smooth skin layer is on the outer surface (Fig. 2H) 12. HPTC performance is compromised on this material in the absence of coating 12.

**Fig. 3:**
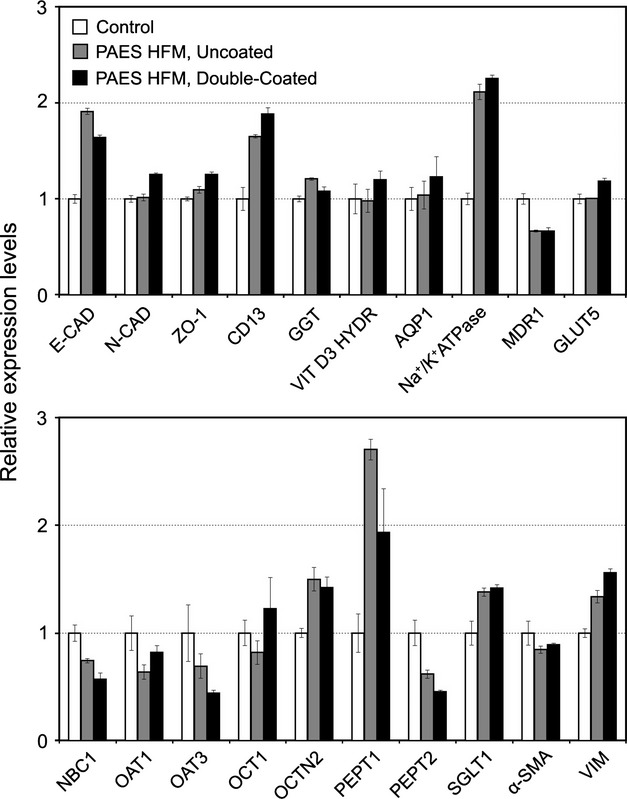
Marker gene expression determined by qPCR. The relative expression levels of the genes indicated (*x*-axis) were assessed with HPTC that were cultivated for 3 days on uncoated (grey bars) or double-coated (black bars) commercial HFM consisting of PAES. The bars show the mean ± SD (*n* = 3) and the mean values obtained with HPTC cultivated on TCPS (control) were set to 1 (white bars).

Human primary renal proximal tubular cells performance on synthetic membranes is usually improved by coatings, but cell growth and survival were not obviously negatively affected on the outer surfaces of uncoated commercial HFM. To address potential, more subtle effects on cell performance we assessed the effects on gene expression patterns (Fig. 3). The expression levels of 20 marker genes were determined by qPCR. This assay is well established and has been applied before to characterize HPTC performance 12, 14, 19. Genes coding for the following epithelial and HPTC-specific markers were included here: E-cadherin (E-CAD), N-cadherin (N-CAD), zonula occludens 1 (ZO-1), aminopeptidase N (CD13), GGT, 25-hydroxyvitamin D_3_ 1α-hydroxylase (VIT D3 HYDR), aquaporin-1 (AQP1), Na^+^/K^+^ ATPase, multidrug resistance gene 1 (MDR1), glucose transporter 5 (GLUT5), Na^+^HCO_3_^−^ co-transporter 1 (NBC1), organic anion transporter 1 (OAT1), OAT3, organic cation transporter 1 (OCT1), organic cation/carnitine transporter 2 (OCTN2), proton-coupled peptide transporter 1 (PEPT1), PEPT2 and sodium-dependent glucose co-transporter 1 (SGLT1). We also determined expression levels of the myofibroblast marker α-smooth muscle actin (α-SMA) and the mesenchymal cell marker vimentin (VIM).

Human primary renal proximal tubular cells were grown on commercial PAES HFM, which remained uncoated or were double coated with DOPA and collagen IV. A double coating consisting of DOPA/collagen IV supports HPTC performance on synthetic membranes better than coatings consisting of a single extracellular matrix protein 13. Control cells were grown on tissue culture plastic (TCPS), which is an excellent substrate for HPTC 14. Some differences in gene expression patterns were observed when cells were grown either on uncoated PAES HFM or on TCPS, which indicated some impact of the different substrates on cell performance. However, in most cases, the expression levels of epithelial and HPTC-specific markers on PAES HFM were at least as high as in the positive control (Fig. 3). This result was in agreement with the previous findings (see above) that overall HPTC performance was not compromised when cells resided on uncoated PAES HFM. Double coating did not improve marker gene expression. Together, these results suggested that the uncoated outer surfaces of commercial HFM were suitable substrates for HPTC and that HPTC performance could not be further improved by applying a coating to these surfaces.

### HPTC performance in bioreactors

**Fig. 4:**
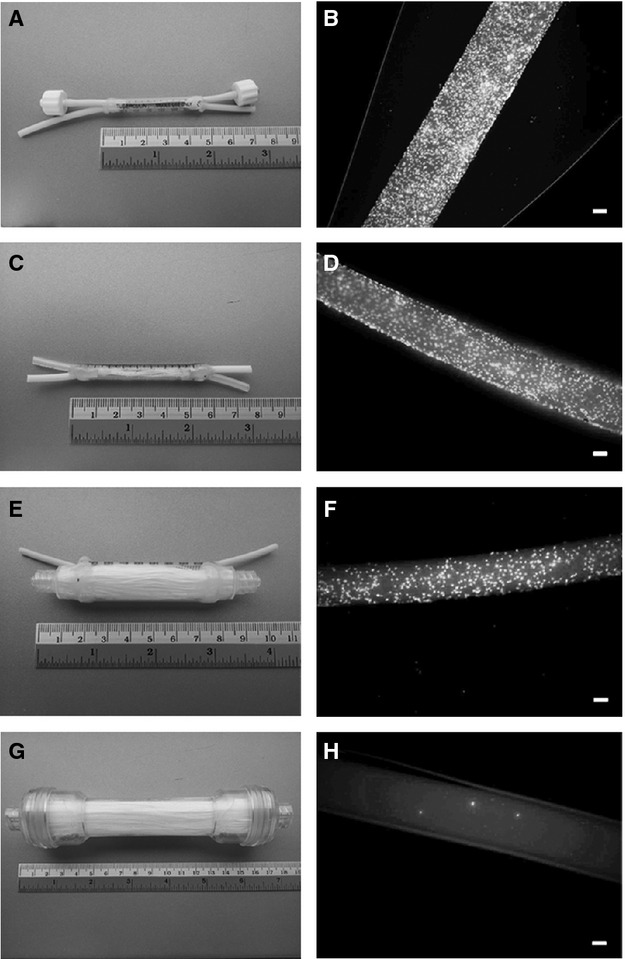
HPTC growth in bioreactors. The bioreactors are shown on the left-hand panels (**A**, **C**, **E** and **G**). All bioreactors contained uncoated commercial PAES HFM derived from the haemofilter of the PrismafleX HF20 set (Gambro). The bioreactors contained one single HFM (A), 25 HFM (C) or ∼250–300 HFM (E). In addition, the unmodified haemofilter from the PrismafleX HF20 set (Gambro) comprising ∼2600 HFM was used (G). The right-hand panels (**B**, **D**, **F** and **H**) show single HFM derived from the bioreactors shown on the left after HPTC had been seeded on the outer HFM surfaces and cultivated for 7 days. Cell nuclei were stained with DAPI and appear as white spots on the grey scale images. HPTC formed confluent monolayers when home-made bioreactors were used (B, D and F). In contrast, only few cells survived in the commercial cartridge with densely packed HFM (H). Ruler in A, C, E and F: cm (white scale) and inch (grey scale); scale bars in B, D, F and H: 100 μm (HFM were squeezed and flattened for epifluorescence microscopy).

Next, we mounted uncoated and unmodified PAES HFM into cartridges and tested HPTC performance on the outer surface under bioreactor conditions. As the work was based on primary cells we started with small cartridges containing 1 single HFM (Fig. 4A). HPTC were seeded on the outer HFM surfaces and perfusion of the extra-HFM space started 8 hrs after seeding with a flow rate of 80 μl/min. A confluent epithelium was obtained on the HFM after 7 days (Fig. 4B). As the result was positive we used a larger bioreactor containing 25 HFM for up-scaling (Fig. 4C). Again, a confluent epithelium was obtained after 7 days (Fig. 4D). After obtaining this result, we seeded the cells into the commercial cartridge (haemofilter from PrismafleX HF20 set, Gambro) containing ∼2600 HFM (Fig. 4G) with a packing density of ∼683 HFM/cm^2^. Here, the outer surfaces of the HFM were largely devoid of cells after 7 days (Fig. 4H). As the previous experiments had indicated that there were no problems with the HPTC-compatibility of the material, we assessed whether the dense packing of HFM in the commercial cartridge was the reason for compromised HPTC performance. Therefore, the next series of experiments was performed with a cartridge that contained ∼250–300 commercial HFM with reduced packing density (∼160 HFM/cm^2^) (Fig. 4E). Under these conditions again a confluent epithelium was obtained after 7 days (Fig. 4F), confirming that indeed dense HFM packing in the commercial cartridge compromised HPTC performance.

Interestingly, the porcine proximal tubular cell line LLC-PK1 (Lewis lung cancer – porcine kidney 1) was less sensitive to the packing density of the HFM and these cells formed confluent epithelia on the outsides of the uncoated HFM also in the commercial cartridge (Supporting Information, Figure S1). It is worth mentioning that cell seeding and the assessment of cell performance is greatly facilitated when the cells are grown on the outer surface of the HFM. This is another advantage of the set-up used here.

### Organic anion transport and back-leak of urea and creatinine

All the following experiments were performed with medium-sized cartridges containing 25 HFM. The extra-HFM space was perfused with a flow rate of 80 μl/min. Where indicated, also the luminal space was perfused at the same rate. We confirmed by immunostaining that the epithelia formed on the outsides of HFM were well differentiated and expressed characteristic marker proteins and brush border enzymes (Supporting Information Figure S2). The results showed that most of the cells expressed the brush border enzyme CD13 (aminopeptidase N), the baso-lateral glucose transporter 1 (GLUT1), the sodium-dependent glucose co-transporter 2 (SGLT2) and the urothelial glycoprotein 10 (URO10). In addition, HPTC in the bioreactor cartridge showed high activity of the brush border enzyme GGT (Supporting Information Figure S2).

**Fig. 5:**
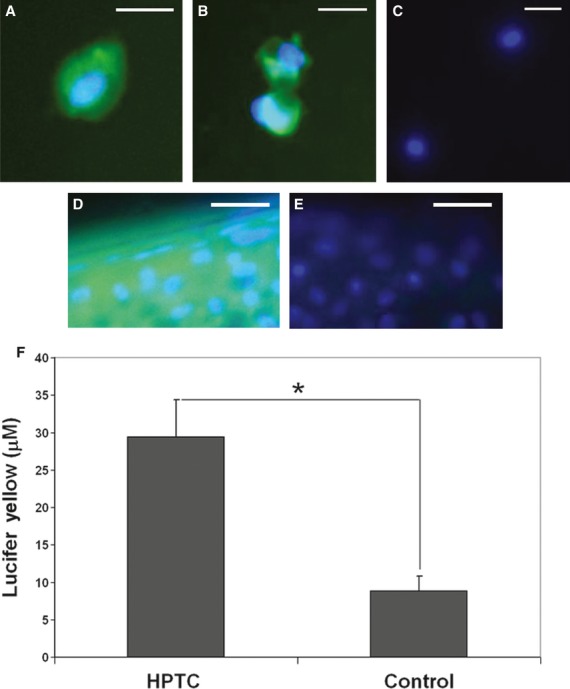
Baso-lateral cellular uptake and transport of lucifer yellow. HPTC were grown on the outer surfaces of PAES HFM in 25-HFM bioreactors. HFM lumina where perfused with 80 μM of lucifer yellow. The extra-HFM space was perfused with cell culture medium not containing lucifer yellow. (**A**, **B** and **C**) show HPTC that were detached from HFM derived from bioreactors that were perfused (A and B) or not perfused (C) with lucifer yellow (green; cell nuclei: blue). Panels (**D**) and (**E**) show HPTC epithelia on the outer surfaces of HFM. The HFM were derived from bioreactors that were perfused (D) or not perfused (E) with lucifer yellow. Scale bars: A, B and C: 25 μm; D and E: 50 μm. The bars in (F) display the concentrations of lucifer yellow (mean ± SD; *n* = 3) in the extra-HFM space, after the luminal spaces had been perfused with lucifer yellow for 24 hrs. The bioreactors contained HPTC or did not contain any cells (control). Significant differences are marked with an asterisk.

To test baso-lateral uptake and transport of organic anions, the luminal spaces of the HFM were perfused for 24 hrs with medium containing 80 μM of lucifer yellow at day 8 after cell seeding. The extra-HFM space was perfused with cell culture medium not containing lucifer yellow. Green fluorescence in the cytoplasm of HPTC residing on the outer surface of the HFM confirmed baso-lateral uptake (Fig. 5D). To exclude the assumption that the green fluorescence observed was because of adsorption of lucifer yellow to the membrane, the cells were detached from some HFM after perfusion with lucifer yellow. Single detached cells showed green fluorescence in the cytoplasm, in contrast to control cells from bioreactors that were not perfused lucifer yellow (Fig. 5A–C).

Transport of lucifer yellow was addressed by measuring the concentration of lucifer yellow in the extra-HFM space. When the outer surfaces of the HFM were covered by epithelia formed by HPTC, a significantly higher concentration of lucifer yellow was measured in the extra-HFM space in comparison to control bioreactors devoid of cells (Fig. 5F). The concentration of lucifer yellow in the extra-HFM space was 3.3-fold higher (difference between the mean values) in cell-containing bioreactors in comparison to the controls. Together, the results demonstrated baso-lateral uptake and transport of lucifer yellow by the epithelium covering the HFM.

**Fig. 6:**
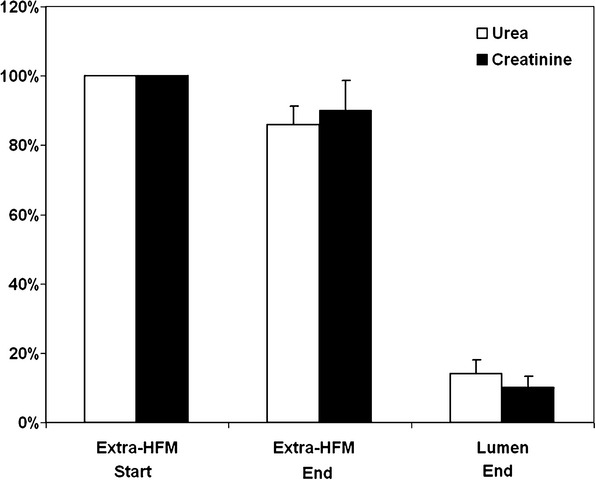
Back-leak of urea and creatinine. The extra-HFM space of 25-HFM bioreactors was perfused for 4 hrs with cell culture medium containing urea and creatinine, whereas the lumina were perfused with medium not containing these compounds. The bars (mean ± SD; *n* = 3) show the relative percentages of urea and creatinine (initial concentrations in the extra-HFM space 100%) in the extra-HFM space at the start of the experiments and at the end after 4 hrs of perfusion. The right-hand bars show the relative percentages of urea and creatinine in the luminal spaces after the 4-hr perfusion period.

After demonstrating transport from the luminal (blood compartment) to the extra-HFM (waste) compartment, it was important to address whether there would be back-leak of waste compounds to the luminal compartment. In a BAK, the waste compartment would contain the haemofiltrate with high concentrations of urea and creatinine. To address the leakiness of the epithelium for these compounds, the extra-HFM space was perfused at day 8 after cell seeding for 4 hrs with medium containing 2 mg/ml of urea and 0.1 mg/ml of creatinine. About 9% (creatinine) and 15% (urea) of these compounds had leaked into the luminal compartment during a time period of 4 hrs (Fig. 6). Previous studies measured during eightfold shorter time periods of 30 min. leakage rates of ∼15% (urea and creatinine, day 7–17 after cell seeding) 21 and <10% (inulin, at least 14 days after seeding) 22.

### Release of interleukins

It is believed that the positive effects of BAK treatment are, at least in part, because of immunomodulatory functions of the device. In animal experiments and clinical trials significant changes in the serum or plasma concentrations of pro- and anti-inflammatory cytokines were observed in the BAK-treated groups 1, 2, 5–8. This included changes in the levels of interleukin-6 (IL-6) and IL-10. It was less obvious whether cytokines were directly released from the BAK and what the contributions of other cell types in the body were. Here, we studied release of IL-6, IL-8 and IL-10 from the bioreactor after perfusion with 10 μg/ml of LPS for 24 hrs or after perfusion with normal medium not containing LPS. LPS administration was performed in two of the previous *in vivo* studies where changes in cytokine levels were observed 1, 7 and one study 2 used *E. coli* administration. The conditions under which the other *in vivo* studies 5, 6 and the clinical trial 8 were performed could not be mimicked *in vitro*.

**Fig. 7:**
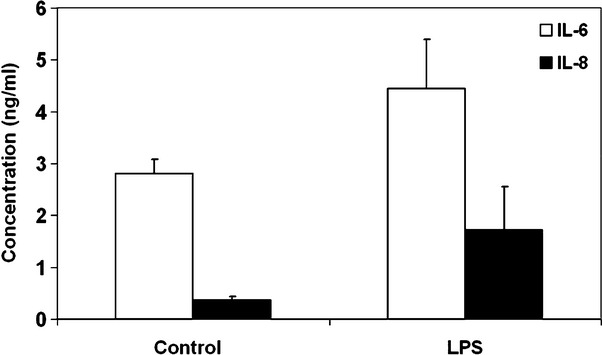
Cytokine production. 25-HFM bioreactors were perfused with cell culture medium containing 10 μg/ml LPS (right-hand bars), or were perfused with normal cell culture medium not containing LPS (control). The levels of IL-6, IL-8 and IL-10 were measured by ELISA and the bars (mean ± SD; *n* = 3) display the concentrations of these interleukins (the levels of IL-10 were always below the detection limit). The levels of IL-6 (white bars) and IL-8 (black bars) were not changed significantly after 24 hrs of LPS stimulation.

Measurements performed by ELISA revealed that the bioreactor released already under normal conditions in the absence of LPS significant amounts of IL-6 and IL-8 (Fig. 7). In particular, high amounts of IL-6 were produced that were in the range of 3 ng/ml. Normal circulating IL-6 levels in humans are ∼2–3 orders of magnitude lower and in the range of ∼1–30 pg/ml 23. The levels of IL-6 and IL-8 were elevated after LPS treatment and the differences between the mean values of the results obtained in the absence or presence of LPS were 1.6-fold (IL-6) and 4.6-fold (IL-8) respectively. However, these changes were not found to be significant and the standard deviations in the presence of LPS were relatively large (Fig. 7). Under all conditions, the amounts of IL-10 were below the detection limit.

### Exposure to ultrafiltrate and relevant perfusion rates do not compromise HPTC

In a last series of experiments, we applied conditions that mimicked the conditions in the bioreactor during animal experiments and clinical trials. At day 8 after cell seeding, the 25-HFM bioreactor was perfused with human ultrafiltrate instead cell culture medium. The ultrafiltrate had been prepared from inactivated human serum. Perfusion with ultrafiltrate was performed for 16 hrs. The flow rate of the ultrafiltrate in the 25-HFM cartridge was 0.5 ml/min. This would correspond to a flow rate of 10 ml/min. in a larger paediatric cartridge (*e.g*. haemofilter from PrismafleX HF20 set, Gambro).

**Fig. 8:**
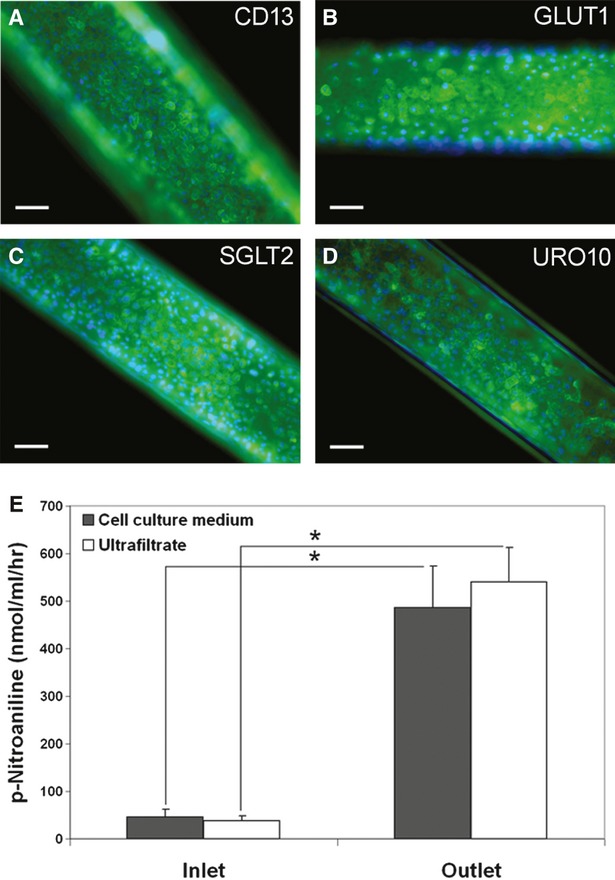
HPTC performance after perfusion with human ultrafiltrate at a relevant flow rate. The extra-HFM space of 25-HFM bioreactors was perfused for 16 hrs with human ultrafiltrate at a flow rate of 0.5 ml/min. Panels (**A**–**D**) show expression of CD13, GLUT1, SGLT2 and URO10 detected by immunofluorescence (green) in HPTC epithelia on the outer HFM surfaces after the 16-hr perfusion period (cell nuclei: blue). The epithelia remained confluent and displayed a normal morphology. Scale bars: 100 μm. The bars in (**E**) show the amounts of p-nitroaniline (mean ± SD; *n* = 3), which is generated in a reaction catalysed by the brush border enzyme GGT. The amounts of p-nitroaniline were measured at the inlet and outlet ports of the bioreactors. The differences between the mean values (inlet and outlet port) were 10.6-fold when cell culture medium was used and 14.2-fold in the presence of ultrafiltrate. The differences in the amounts of p-nitroaniline measured at the inlet and outlet ports under the respective conditions were significant (indicated by asterisks). Perfusion of the bioreactor with ultrafiltrate at a flow rate of 0.5 ml/min. (white bars) did not significantly alter the amounts of p-nitroaniline in comparison to perfusion with cell culture medium at a flow rate of 80 μl/min. (black bars).

The result showed that after perfusion with ultrafiltrate at 0.5 ml/min a confluent epithelium with a similar morphology and marker expression pattern was present (Fig. 8) as observed during the previous experiments (compare Supporting Information Figure S2). Furthermore, perfusion with ultrafiltrate and higher flow rates did not affect GGT activity (Fig. 8B, see also Supporting Information Figure S2). Together, the results suggested that a robust and functional epithelium was formed by HPTC on the outer surfaces of unmodified commercial HFM that was not negatively affected by conditions mimicking the situation during large animal experiments and clinical trials.

## Discussion

Here, we describe a novel BAK design. One major feature of the novel design is a reversed architecture of the bioreactor in comparison to the current predominantly used BAK design that has been applied now for more than a decade 1–10, 24–26. In the configuration described in our current study, HPTC are grown on the outer non-haemocompatible HFM surfaces, whereas the blood is always exposed to the luminal haemocompatible surfaces. This helps to avoid problems with HFM pore clogging 12 and improves haemocompatibility, which is of utmost importance in a BAK. Furthermore, applying this configuration allows the use of unmodified commercial HFM for HPTC growth.

Some earlier studies suggested or applied bioreactors where renal cells were also seeded on the outer surfaces of HFM 27–30. Probably some of the specific advantages of using this configuration were not obvious in these studies, and this might be one reason why this configuration was not widely adopted. For instance, Ip and Aebischer 27 suggested that the cells be seeded either on the outer or on the inner HFM surfaces. As the animal cell lines used by these authors are indeed much less selective than HPTC in terms of growth substrates 10 it makes indeed no difference on which kind of surface these cells are grown. In other studies performed with a rabbit cell line, a collagen coating was applied to the outer HFM surfaces 29, 30.

Our results showed that HPTC formed differentiated epithelia on the uncoated outer surfaces of commercial HFM consisting of either PSF or PAES. The epithelia expressed characteristic marker proteins and brush border enzymes, and the state of cell differentiation could not be further improved by applying a coating. In addition, the results showed that the epithelia showed baso-lateral uptake and transport of the organic anion lucifer yellow from the blood compartment into the waste compartment. The epithelia also prevented back-leak of urea and creatinine into the blood compartment. They remained confluent and well differentiated when exposed to human ultrafiltrate at relevant flow rates. Altogether, this showed that unmodified and uncoated outer surfaces of commercial HFM are a suitable substrate for HPTC under bioreactor conditions. Using uncoated HFM is less costly and helps to avoid batch-to-batch variability, in particular with regard to approaches where extracellular matrix proteins derived for natural sources are used 1, 4, 8, 9. In our experience, HPTC performance is also more reliable when a HPTC-compatible material is used that does not require further modifications to achieve HPTC growth and survival.

An interesting question is why HPTC perform better on the outer surface of HFM than on the smooth and haemocompatible skin layer. An obvious difference between these distinct surfaces is their topology, which could impact HPTC performance. This impact might be indirect and mediated by increased protein adsorption to the rough surface 12. Indeed, a hallmark of haemocompatible surfaces is their reduced adhesiveness for proteins and cells. Our previous studies showed that HPTC perform particularly well on adhesive substrates like DOPA-coated surfaces or membranes consisting of PSF-Fullcure 13. Notably, the haemocompatibility of such surfaces, which sustain HPTC performance, is very low with extensive platelet adhesion and activation (Zay Yar Oo, unpublished observation). Thus, as HPTC-compatible surfaces appear to be non-haemocompatible, and *vice versa*, it appears to be most straightforward to use in BAK bioreactors the haemocompatible membrane surface for the blood compartment and the non-haemocompatible surfaces for the cell compartment. Bioreactors with a respective reversed architecture, as described here, would not mimic the architecture of the renal tubule, which is also not required.

Another advantage of growing the cells on the outer surfaces of HFM is that cell performance can be easily monitored. No sectioning of the tiny HFM is required for visual monitoring and large numbers of HFM can be rapidly assessed. Furthermore, cell seeding is greatly facilitated and sheer stress during injection of the cells into the narrow HFM lumen is avoided. HPTC are particularly sensitive to mechanical stress. This was also demonstrated here by the fact that HPTC did not survive on the densely packed HFM in the commercial cartridge, whereas the performance of the porcine proximal tubular cell line LLC-PK1 was not compromised under these conditions.

Given the high sensitivity of HPTC to mechanical stress, it is also important to avoid transmembrane pressure 16, 17. As the flow rate of the haemofiltrate is always much lower than the flow rate of the blood, transmembrane pressure is always generated in the bioreactor unit when all or substantial parts of the blood exiting the haemofilter flow into the bioreactor 1–9. Therefore, we propose to use similar flow rates of the blood and the haemofiltrate, which would prevent the generation of transmembrane pressure. The size of the bioreactor unit should be adjusted accordingly. The use of normal paediatric cartridges might not be possible because of the dense packing of HFM, and the use of cartridges with a lower density of HFM would be recommendable. In case of problems with too low flow rates and too small membranes surface areas in the bioreactor part of the processed haemofiltrate could be recycled and pumped back from the waste reservoir to the haemofiltrate reservoir. This would allow to increase the flow rates in the haemofiltrate and the blood compartment in the bioreactor, and to increase the size of the bioreactor. Furthermore, this would increase exposure of the haemofiltrate to the cells, which might improve processing. Alternatively, replacement fluid could be added to the haemofiltrate entering the bioreactor, which would also allow an increase in the flow rates in the bioreactor. Both methods for increasing the flow rates could be combined.

When the flow rates of the haemofiltrate and the blood in the bioreactor would be similar, major parts of the blood exiting the haemofilter could not enter the bioreactor and would flow back into the animal or patient. This would further improve haemocompatibility, as most of the blood would not be exposed to both cartridges.

An open question is whether the efficacy of BAK treatment would be compromised under these conditions, where less blood would be exposed to the bioreactor (it should be noted that we proposed several changes and the reversed bioreactor design discussed above is unrelated to this feature). What contributes to the current uncertainties is the fact that it is not known why BAK treatment improved survival of critically ill patients with AKI 9 and also improved survival in respective animal models 2, 5–7. Reabsorption functions of the proximal tubular cells, for which larger cell numbers would be required 25, would not be expected to play a major role in this situation 1. It has been speculated that immunomodulatory functions of the BAK are important. Some results suggested that in particular the levels of IL-6 and IL-10 play a role 1, 2, 5, 6–8. As previous studies measured the serum or plasma levels of cytokines during BAK treatment it remained unclear whether and how cytokine levels were influenced by the cells in the bioreactor.

Here, we examined the levels of IL-6, IL-8 and IL-10 produced in the bioreactor. Although the levels of IL-10 were always below the detection limit, significant amounts of IL-6 and IL-8 were released by the bioreactor. In particular, high levels of IL-6 were produced and the concentrations measured here were ∼100- to 1000-fold higher than normal circulating IL-6 levels in humans. Given these results, it would be indeed expected that BAK treatment would have immunomodulatory effects, as observed in previous studies 1, 2, 5–8, although it would have to be determined in detail which fractions of cytokines produced by the bioreactor would enter the blood compartment under the actual conditions applied Although HPTC in the bioreactor do not appear to produce IL-10, it has been observed after injection of recombinant human IL-6 into healthy volunteers that the levels of two anti-inflammatory cytokines, including IL-10, were increased by IL-6 31. Such effects of IL-6 could probably explain the increased levels of IL-10 in animals during BAK treatment 1, 2. Typically, IL-6 acts at distant sites and the effects of BAK treatment on cytokine expression patterns in peripheral blood mononuclear cells might be also explained by such effects 7. It is not clear why HPTC produced high IL-6 levels under normal conditions, but we observed this phenomenon consistently (Yao Li and Farah Tasnim, unpublished results). Probably this reflects a reaction of the cells to the *in vitro* conditions, which are very different from the normal *in vivo* environment.

Here, we observed no significant increase in the levels of IL-6 and IL-8 after prolonged exposure to LPS for 24 hrs. These results are in agreement with a previous study that found no significant effects of LPS on the levels of IL-6 produced by HPTC (called PTEC in this study) 32. The results of this study and other results obtained by us show that HPTC indeed significantly increase secretion of IL-6 and IL-8 in response to other stimuli, such as exposure to IL-1α 32 or different kinds of insults (Yao Li and Daniele Zink, unpublished results). However, HPTC appear to be only minimally responsive to LPS, which has been applied in BAK-related animal studies 1, 7. Nevertheless, although no direct effect of LPS on HPTC has been found, indirect effects on cytokine production by HPTC during animal experiments cannot be excluded. In future, it would be important to further investigate cytokine release by BAK and the cross-talk of HPTC in the bioreactor with other cell types in the body under normal and disease conditions. This would be essential for understanding the effects of BAK treatment.

## Supporting information

**Figure S1** LLC-PK1 cells were cultivated with perfusion in the unmodified haemofilter from the PrismafleX HF20 set (Gambro) shown in (a).

**Figure S2** Expression of marker proteins and brush border enzymes.
