# Supplementary material for: A novel design of bioartificial kidneys with improved cell performance and haemocompatibility
Source: J Cell Mol Med. 2013 Mar 11;17(4):497–507. doi: 10.1111/jcmm.12029 (PMC3822650; doi:10.1111/jcmm.12029)
Supplement: Supplementary file 1 [file jcmm0017-0497-SD1.docx]

**Supplementary Data**

**A novel design of bioartificial kidneys with improved cell performance and haemocompatibility**

Zay Yar Oo, Karthikeyan Kandasamy, Farah Tasnim and Daniele Zink^*^

Institute of Bioengineering and Nanotechnology, 31 Biopolis Way, The Nanos, Singapore 138669, Singapore

* Corresponding author:

Phone: +65 6824 7107, Fax: +65 6478 9080, E-mail addresses: [dzink@ibn.a-star.edu.sg](mailto:dzink@ibn.a-star.edu.sg)

**Supplementary Figure Legends**

**Supplementary Figure S1:** LLC-PK1 cells were cultivated with perfusion in the unmodified haemofilter from the PrismafleX HF20 set (Gambro) shown in (a). (b) shows the LLC-PK1 epithelium on the outer surface of an HFM removed from the hemofilter after 7 days. DAPI-stained cell nuclei appear as white spots. Ruler in a: cm (white scale) and inch (grey scale); scale bar in b: 100 μm (the HFM was squeezed and flattened for epifluorescence microscopy).

**Supplementary Figure S2:** Expression of marker proteins and brush border enzymes. HPTC were cultivated in 25-HFM bioreactors. Panels (a-d) show confluent epithelia of HPTC on the outer surfaces of PAES HFM. CD13, GLUT1, SGLT2 and URO10 were detected by immunofluorescence (green; cell nuclei: blue). Scale bars: 100 μm. The bars in (e) show the amounts of p-nitroaniline (mean +/- s.d.; n = 3), which is generated in a reaction catalyzed by the brush border enzyme GGT. The amounts of p-nitroaniline were measured at the inlet and outlet ports of the bioreactors. Significantly higher amounts (indicated by an asterisk) were measured at the outlet ports in comparison to the inlet ports when the bioreactors contained HPTC (black bars). Similar bioreactors containing NIH 3T3 fibroblasts were used as control (white bars) and here the amounts of p-nitroaniline were always very low.
