# Supplementary figures and images for: A novel design of bioartificial kidneys with improved cell performance and haemocompatibility
Source: J Cell Mol Med. 2013 Mar 11;17(4):497–507. doi: 10.1111/jcmm.12029 (PMC3822650; doi:10.1111/jcmm.12029)

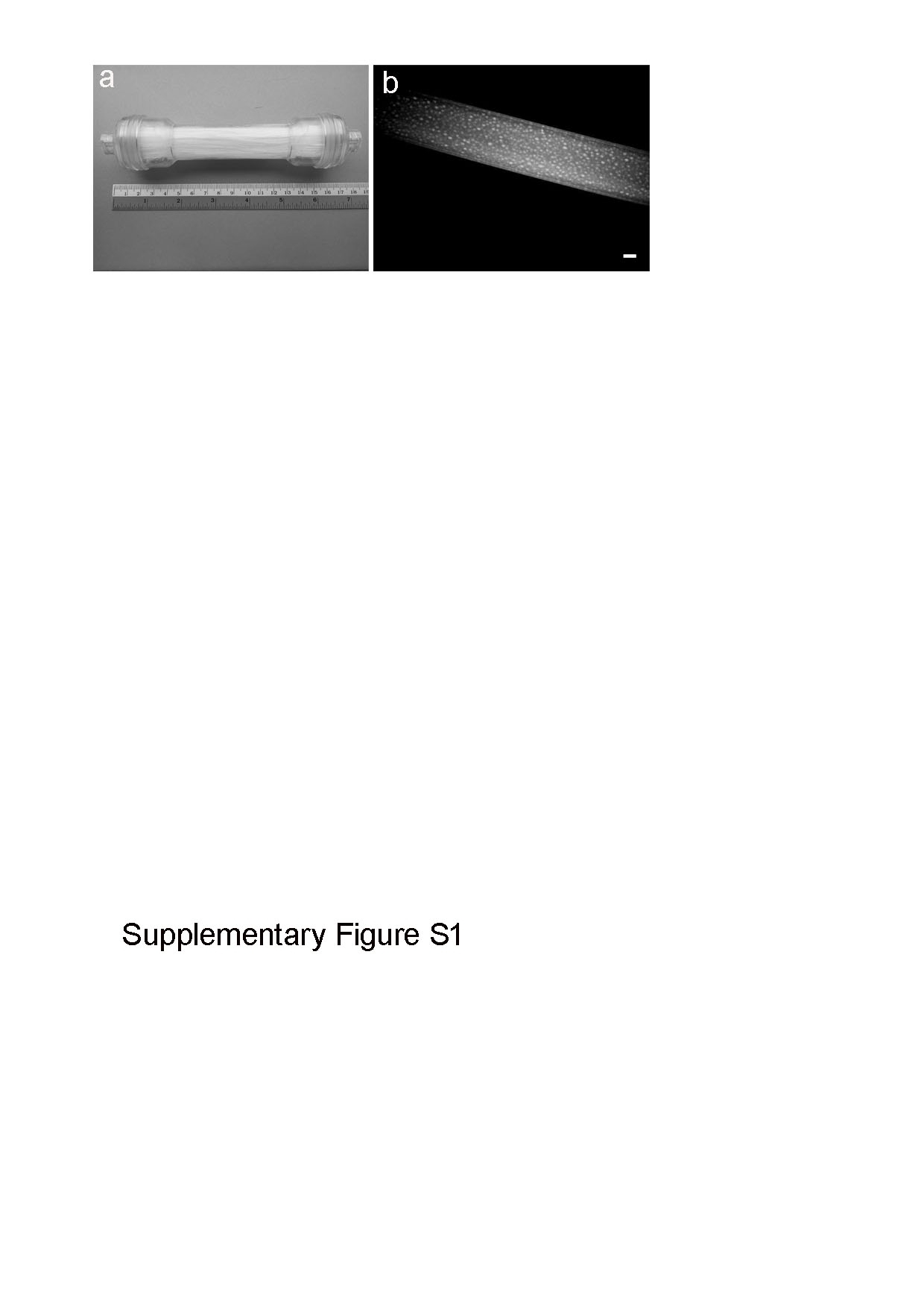

Supplement: Supplementary file 2 [file jcmm0017-0497-SD2.jpg]

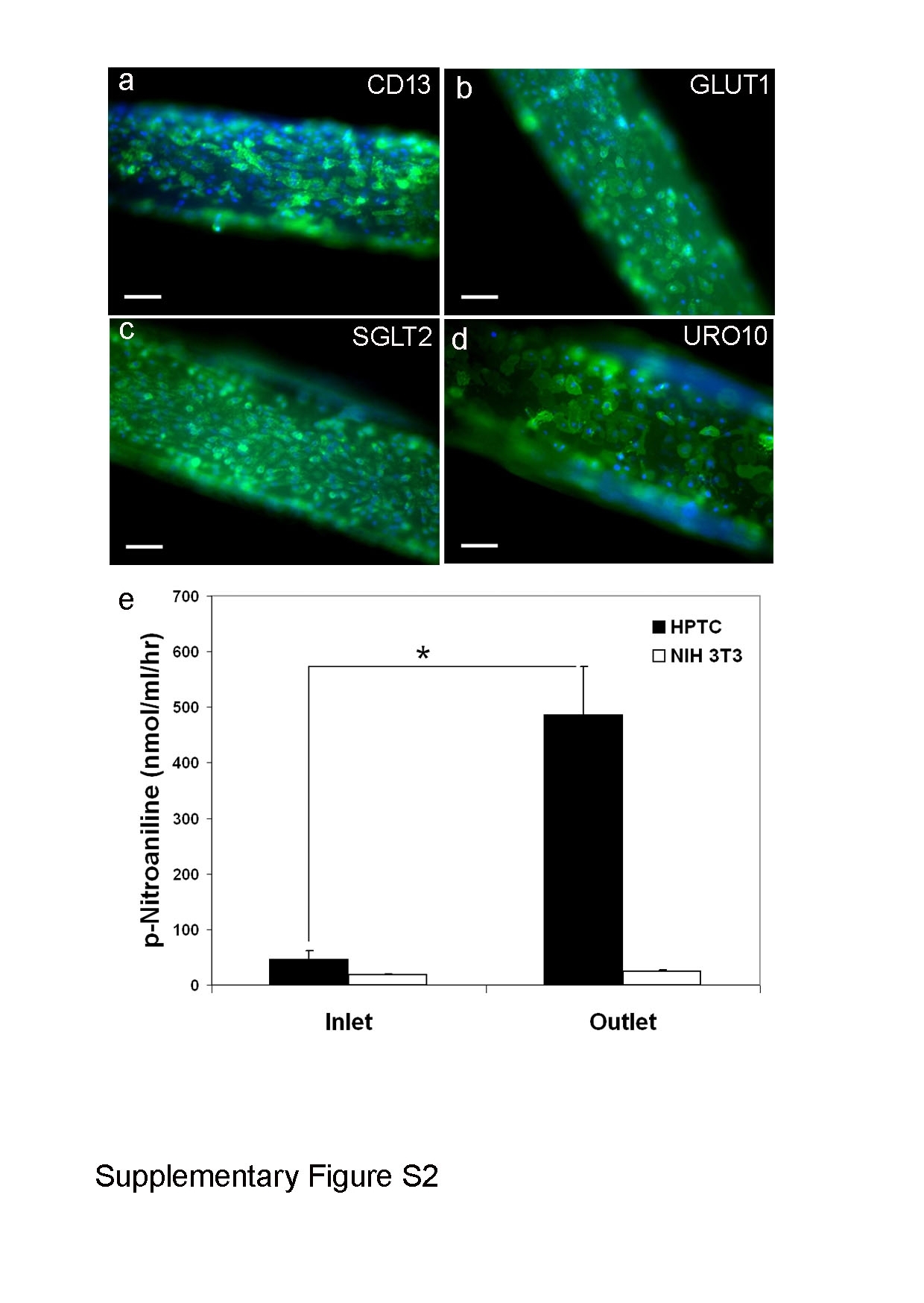

Supplement: Supplementary file 3 [file jcmm0017-0497-SD3.jpg]
